# 1-(5,5-Di­meth­oxy­pent­yl)-3-methyl­imidazolium-2-carboxyl­ate

**DOI:** 10.1107/S1600536813027013

**Published:** 2013-10-05

**Authors:** Olaf Walter

**Affiliations:** a IKFT, KIT-Campus Nord, Hermann-von-Helmholtz-Platz 1, 76344 Eggenstein-Leopoldshafen, Germany

## Abstract

The title compound, C_12_H_20_N_2_O_4_, represents one example of a zwitterionic imidazolium salt with a carboxyl­ate group at the 2-position of the imidazolium ring. The dihedral angle between the heterocyclic ring and the carboxyl­ate group is 31.3 (1)°. The side chain linking the N atom of the ring and the methine C atom has a *gauche*–*anti*–*anti* conformation [torsion angles = −60.3 (2), −175.7 (2) and 178.7 (2)°, respectively]. In the crystal, mol­ecules are linked by short C—H⋯O hydrogen bonds involving the C—H groups in the aromatic ring to generate (001) sheets.

## Related literature

For related zwitterionic structures, see: Gurau *et al.* (2011 ▶); Holbrey *et al.* (2003 ▶); Smiglak *et al.* (2007 ▶); Reichert *et al.* (2010 ▶).
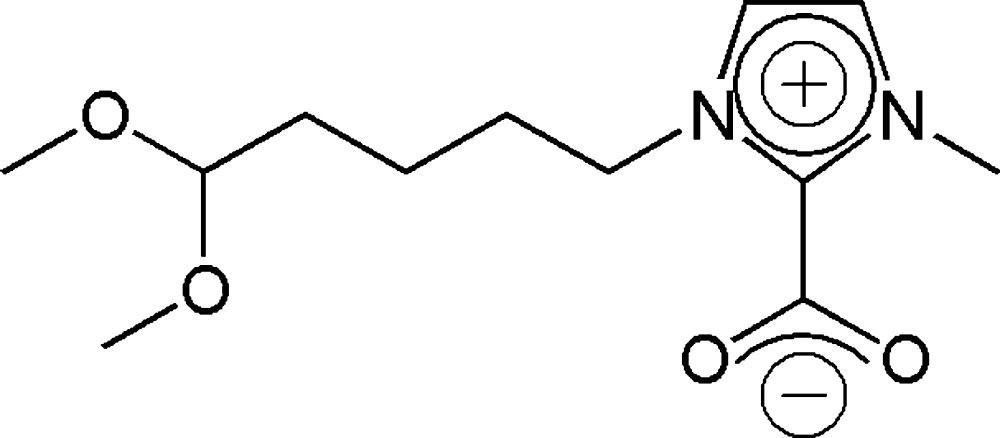


## Experimental

### 

#### Crystal data

C_12_H_20_N_2_O_4_
*M* *_r_* = 256.30
Triclinic, 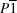

*a* = 7.1943 (8) Å
*b* = 7.3259 (8) Å
*c* = 13.2263 (15) Å
α = 85.124 (2)°
β = 85.542 (2)°
γ = 72.938 (2)°
*V* = 662.98 (13) Å^3^
*Z* = 2
Mo *K*α radiation
μ = 0.10 mm^−1^
*T* = 200 K
0.3 × 0.15 × 0.15 mm

#### Data collection

Siemens SMART CCD 1000 diffractometer
Absorption correction: multi-scan (*SADABS*; Bruker, 1997 ▶) *T*_min_ = 0.940, *T*_max_ = 1.000
8022 measured reflections
3192 independent reflections
1962 reflections with *I* > 2σ(*I*)
*R*_int_ = 0.023

#### Refinement

*R*[*F* ^2^ > 2σ(*F* ^2^)] = 0.054
*wR*(*F* ^2^) = 0.178
*S* = 1.03
3192 reflections
171 parameters
Only H-atom displacement parameters refined
Δρ_max_ = 0.27 e Å^−3^
Δρ_min_ = −0.32 e Å^−3^

### 

Data collection: *SMART* (Bruker, 1997 ▶); cell refinement: *SMART*; data reduction: *SAINT* (Bruker, 1997 ▶); program(s) used to solve structure: *SHELXS97* (Sheldrick, 2008 ▶); program(s) used to refine structure: *SHELXL2013* (Sheldrick, 2013 ▶); molecular graphics: *XPMA* (Zsolnai, 1996 ▶) and *ORTEP-3 for Windows* (Farrugia, 2012 ▶); software used to prepare material for publication: *publCIF* (Westrip, 2010 ▶).

## Supplementary Material

Crystal structure: contains datablock(s) I. DOI: 10.1107/S1600536813027013/hb7143sup1.cif

Structure factors: contains datablock(s) I. DOI: 10.1107/S1600536813027013/hb7143Isup2.hkl

Supplementary material file. DOI: 10.1107/S1600536813027013/hb7143Isup3.mol

Supplementary material file. DOI: 10.1107/S1600536813027013/hb7143Isup4.cml

Additional supplementary materials:  crystallographic information; 3D view; checkCIF report

## Figures and Tables

**Table 1:** Hydrogen-bond geometry (Å, °)

*D*—H⋯*A*	*D*—H	H⋯*A*	*D*⋯*A*	*D*—H⋯*A*
C3—H3⋯O2^i^	0.93	2.30	3.141 (2)	151
C4—H4⋯O1^ii^	0.93	2.30	3.127 (2)	148

Symmetry codes: (i) 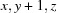; (ii) 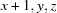.

**Fig. 1.**
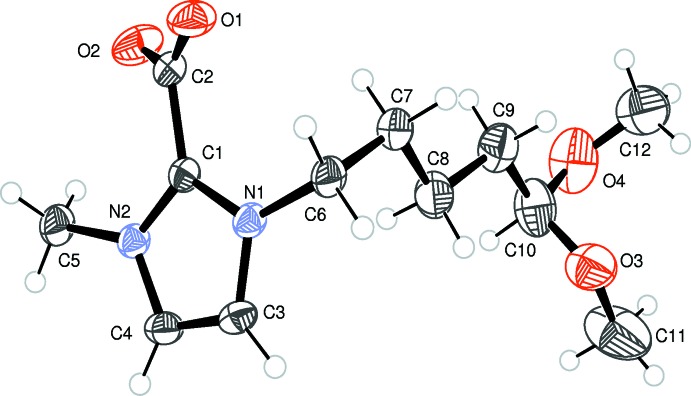
: View to the molecular structure of 1-(5,5-dimethoxypentyl)-3-methyl-imidazolium-2-carboxylate; ellipsoids at 50% probability level

**Fig. 2.**
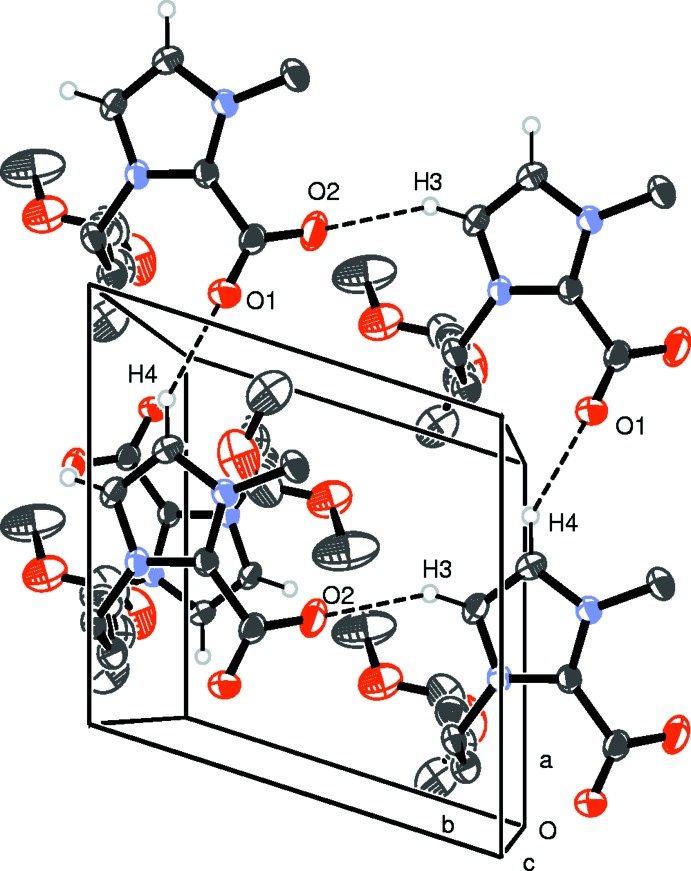
: Visualization of the H-bridge bond system of 1-(5,5-dimethoxypentyl)-3-methyl-imidazolium-2-carboxylate in the crystal; ellipsoids at 50% probability level

**Fig. 3.**
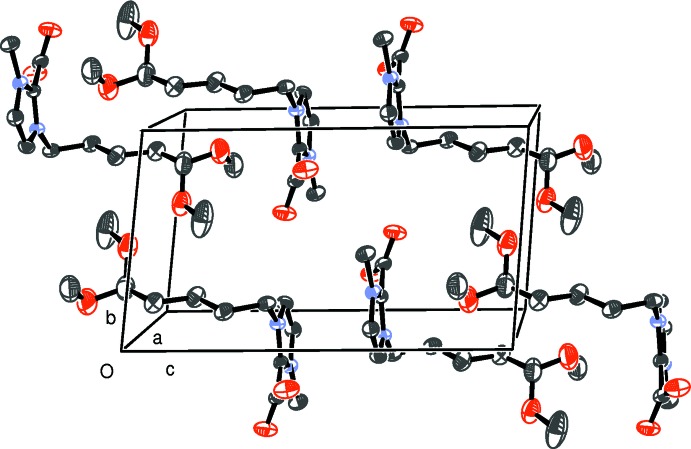
: Molecular arrangement of 1-(5,5-dimethoxypentyl)-3-methyl-imidazolium-2-carboxylate in the cell; ellipsoids at 50% probability level

## supplementary crystallographic information

### 1. Comment

1-(5,5-Dimethoxypentyl)-3-methyl-imidazolium-2-carboxylate is one example for only a few in the literature reported zwitterions consisting of a positively charged imidazolium ring carrying in 2-position a carboxylato group (see Figure 1). The C—O bond distances in the carboxylato group are determined to 1.234 (2) and 1.238 (8) Å and are therefor comparable to those of Holbrey *et al.* (2003), Gurau *et al.* (2011), or Smiglak *et al.* (2007). Within the imidazoliumcarboxylate-unit complete delocalization of the π-electrons is possible. However, the embedding of the carboxylato group of 1-(5,5-dimethoxypentyl)-3-methyl-imidazolium-2-carboxylate in the formation of intermolecular H-bridge bonds (H3···O2 2.30 Å and H4···O1 2.30 Å) causes a distortion from the planarity (torsion angle N1—C1—C2—O1 -29.6 °, see figure 2, 3). The imidazolium rings are placed approximately in the a,b-plane of the elementary cell around the middle of the *c* axis and the center of the cell whereas the in 1-position placed 5,5-dimethoxypentyl-substituent is oriented parallelly to the *c* axis perpendicularly to the imidazole ring monstrating *versus* the next layer of imidazolium rings in the next cell (figure 3). By this arrangement the 5,5-dimethoxypentyl-substituents form an unpolar region in the cell enabling the formation of van-der-Waals interactions to those of neighboured molecules. Another example of a zwitterionic imidazolium based molecule with to different substituents in 1,3-position of the imidazolium unit is reported in Reichert *et al.* (2010). In the reported structure the negative charge equilibrating the positive one of the imidazolium ring is a 3-sulfonatopropyl-group. The charges are separated, due to this and the shortness of the propyl-group the complete molecular arrangment in the cell is different and not comparable. However a comparison of the structural features of the imidazolium ring in 1-(5,5-dimethoxypentyl)-3-methyl-imidazolium-2-carboxylate to those of the other in the literature reported imidazolium based carboxylates show a general and high congruency.

### 2. Experimental

1-(5,5-dimethoxypentyl)-3-methyl-imidazolium-2-carboxylate is obtained from the reaction of 2.0 g (7.8 mmol) 1-(5,5-dimethoxypentyl)-imidazole heated to 120° C in an autoclave for 3 h together with 10 ml of methanol and 10 ml of dimethylcarbonate after removal of the solvent and re-crystallization from small amounts of methanol in 55% yield. 1-(5,5-dimethoxypentyl)-imidazole has been prepared by alkylation of imidazole with 1-chloro-5,5-dimethoxypentane.

### 3. Refinement

The positions of all H atoms are calculated on geometrical positions according to the hybridization of the atoms they are bound to. The isotropic U values of the hydrogen atoms are refined group-wisely.

### Crystal data

**Table:**

C_12_H_20_N_2_O_4_	*Z* = 2
*M**_r_* = 256.30	*F*(000) = 276
Triclinic, *P*1	*D*_x_ = 1.284 Mg m^−^^3^
*a* = 7.1943 (8) Å	Mo *K*α radiation, λ = 0.71073 Å
*b* = 7.3259 (8) Å	Cell parameters from 49 reflections
*c* = 13.2263 (15) Å	θ = 2.8–42.3°
α = 85.124 (2)°	µ = 0.10 mm^−^^1^
β = 85.542 (2)°	*T* = 200 K
γ = 72.938 (2)°	Block, colourless
*V* = 662.98 (13) Å^3^	0.3 × 0.15 × 0.15 mm

### Data collection

**Table:**

Siemens SMART CCD 1000 diffractometer	3192 independent reflections
Radiation source: fine-focus sealed tube	1962 reflections with *I* > 2σ(*I*)
Graphite monochromator	*R*_int_ = 0.023
Detector resolution: 8 pixels mm^-1^	θ_max_ = 28.3°, θ_min_ = 1.6°
ω scan	*h* = −9→9
Absorption correction: multi-scan (*SADABS*; Bruker, 1997)	*k* = −9→9
*T*_min_ = 0.940, *T*_max_ = 1	*l* = −17→17
8022 measured reflections	

### Refinement

**Table:**

Refinement on *F*^2^	Primary atom site location: structure-invariant direct methods
Least-squares matrix: full	Secondary atom site location: difference Fourier map
*R*[*F*^2^ > 2σ(*F*^2^)] = 0.054	Hydrogen site location: inferred from neighbouring sites
*wR*(*F*^2^) = 0.178	Only H-atom displacement parameters refined
*S* = 1.03	*w* = 1/[σ^2^(*F*_o_^2^) + (0.1021*P*)^2^] where *P* = (*F*_o_^2^ + 2*F*_c_^2^)/3
3192 reflections	(Δ/σ)_max_ < 0.001
171 parameters	Δρ_max_ = 0.27 e Å^−^^3^
0 restraints	Δρ_min_ = −0.32 e Å^−^^3^

### Special details

**Table:**

Experimental. Spectroscopic data: ^1^H NMR (CDCl_3_): δ = 6.93, s (br), 1H, CH(arom); 6.91 (br), s, 1H, CH(arom); 4.51, t, ^3^J_HH_= 7.4 Hz, 2H, NCH_2_; 4.26, t, ^3^J_HH_= 5.5 Hz, 1H, COH; 4.06, s (br), 3H, NCH_3_; 3.24, s, 6H, OCH_3_; 1.84, p, ^3^J_HH_= 7.4 Hz, 2H, CH_2_; 1.56, m, 2H, CH_2_; 1.35, m, CH_2_.
Geometry. All e.s.d.'s (except the e.s.d. in the dihedral angle between two l.s. planes) are estimated using the full covariance matrix. The cell e.s.d.'s are taken into account individually in the estimation of e.s.d.'s in distances, angles and torsion angles; correlations between e.s.d.'s in cell parameters are only used when they are defined by crystal symmetry. An approximate (isotropic) treatment of cell e.s.d.'s is used for estimating e.s.d.'s involving l.s. planes.
Refinement. The structure was solved by direct methods and refined to an optimum R_1_ value with SHELXL. The data of the structure have been deposited at the CCDC with the reference number 962824.

### Fractional atomic coordinates and isotropic or equivalent isotropic displacement parameters (Å^2^)

**Table:**

	*x*	*y*	*z*	*U*_iso_*/*U*_eq_	
O1	0.1515 (2)	−0.2046 (2)	0.42195 (13)	0.0450 (4)	
O2	0.3946 (2)	−0.4369 (2)	0.35645 (13)	0.0472 (4)	
O3	0.2709 (3)	0.4306 (3)	−0.04153 (13)	0.0594 (5)	
O4	0.2379 (3)	0.1828 (3)	−0.13125 (14)	0.0745 (6)	
N1	0.3940 (2)	0.0490 (2)	0.36421 (11)	0.0254 (4)	
N2	0.6433 (2)	−0.1977 (2)	0.38760 (11)	0.0252 (4)	
C1	0.4498 (3)	−0.1435 (2)	0.37745 (13)	0.0239 (4)	
C2	0.3182 (3)	−0.2742 (3)	0.38566 (15)	0.0298 (5)	
C3	0.5536 (3)	0.1147 (3)	0.36567 (15)	0.0297 (5)	
H3	0.5545	0.2418	0.3572	0.030 (4)*	
C4	0.7090 (3)	−0.0391 (3)	0.38158 (14)	0.0303 (5)	
H4	0.8366	−0.0377	0.3874	0.030 (4)*	
C5	0.7684 (3)	−0.3936 (3)	0.40763 (17)	0.0357 (5)	
H5A	0.7251	−0.4468	0.4707	0.050 (4)*	
H5B	0.9005	−0.3916	0.4117	0.050 (4)*	
H5C	0.7616	−0.4704	0.3535	0.050 (4)*	
C6	0.1956 (3)	0.1756 (3)	0.34690 (15)	0.0304 (5)	
H6A	0.1940	0.3076	0.3510	0.040 (2)*	
H6B	0.1056	0.1465	0.3999	0.040 (2)*	
C7	0.1284 (3)	0.1521 (3)	0.24391 (16)	0.0374 (5)	
H7A	0.1252	0.0210	0.2416	0.040 (2)*	
H7B	−0.0036	0.2345	0.2374	0.040 (2)*	
C8	0.2543 (3)	0.1980 (3)	0.15374 (17)	0.0438 (6)	
H8A	0.2656	0.3258	0.1580	0.040 (2)*	
H8B	0.3838	0.1089	0.1566	0.040 (2)*	
C9	0.1725 (4)	0.1868 (4)	0.05333 (18)	0.0491 (6)	
H9A	0.0414	0.2734	0.0518	0.040 (2)*	
H9B	0.1635	0.0581	0.0491	0.040 (2)*	
C10	0.2915 (4)	0.2362 (4)	−0.03937 (18)	0.0577 (7)	
H10	0.4288	0.1672	−0.0303	0.042 (6)*	
C11	0.4004 (5)	0.4901 (6)	−0.1162 (2)	0.0980 (13)	
H11A	0.5322	0.4170	−0.1043	0.122 (6)*	
H11B	0.3865	0.6235	−0.1115	0.122 (6)*	
H11C	0.3692	0.4695	−0.1828	0.122 (6)*	
C12	0.0419 (5)	0.2754 (5)	−0.1545 (2)	0.0674 (8)	
H12A	−0.0435	0.2222	−0.1100	0.122 (6)*	
H12B	0.0242	0.2570	−0.2237	0.122 (6)*	
H12C	0.0125	0.4098	−0.1455	0.122 (6)*	

### Atomic displacement parameters (Å^2^)

**Table:**

	*U*^11^	*U*^22^	*U*^33^	*U*^12^	*U*^13^	*U*^23^
O1	0.0251 (8)	0.0351 (9)	0.0739 (12)	−0.0105 (6)	0.0030 (7)	0.0031 (7)
O2	0.0530 (10)	0.0268 (8)	0.0648 (11)	−0.0175 (7)	0.0080 (8)	−0.0088 (7)
O3	0.0608 (12)	0.0811 (14)	0.0435 (10)	−0.0348 (10)	−0.0001 (8)	0.0047 (9)
O4	0.0951 (16)	0.0718 (14)	0.0456 (11)	−0.0059 (12)	−0.0002 (11)	−0.0108 (9)
N1	0.0264 (8)	0.0199 (8)	0.0290 (8)	−0.0056 (6)	−0.0007 (6)	−0.0009 (6)
N2	0.0236 (8)	0.0233 (8)	0.0276 (8)	−0.0058 (6)	−0.0001 (6)	0.0000 (6)
C1	0.0248 (9)	0.0210 (9)	0.0248 (9)	−0.0053 (7)	−0.0010 (7)	−0.0006 (7)
C2	0.0325 (11)	0.0247 (10)	0.0336 (11)	−0.0114 (8)	−0.0063 (8)	0.0051 (8)
C3	0.0342 (11)	0.0243 (10)	0.0341 (11)	−0.0140 (8)	−0.0013 (8)	−0.0026 (8)
C4	0.0285 (10)	0.0331 (11)	0.0327 (11)	−0.0147 (9)	−0.0006 (8)	−0.0019 (8)
C5	0.0301 (11)	0.0263 (11)	0.0453 (13)	−0.0012 (8)	−0.0043 (9)	0.0042 (9)
C6	0.0272 (10)	0.0211 (9)	0.0390 (11)	−0.0015 (8)	−0.0019 (8)	0.0004 (8)
C7	0.0345 (11)	0.0314 (11)	0.0447 (13)	−0.0078 (9)	−0.0075 (9)	0.0046 (9)
C8	0.0398 (13)	0.0454 (13)	0.0421 (13)	−0.0077 (10)	−0.0037 (10)	0.0043 (10)
C9	0.0574 (15)	0.0446 (14)	0.0436 (14)	−0.0128 (12)	−0.0061 (11)	0.0032 (10)
C10	0.0615 (17)	0.0628 (18)	0.0388 (14)	−0.0021 (14)	−0.0043 (12)	−0.0029 (12)
C11	0.079 (2)	0.180 (4)	0.0543 (19)	−0.076 (3)	−0.0008 (16)	0.022 (2)
C12	0.082 (2)	0.082 (2)	0.0481 (16)	−0.0391 (18)	−0.0070 (15)	−0.0028 (14)

### Geometric parameters (Å, º)

**Table:**

O1—C2	1.234 (2)	C6—C7	1.520 (3)
O2—C2	1.238 (2)	C6—H6A	0.9700
O3—C10	1.386 (3)	C6—H6B	0.9700
O3—C11	1.433 (3)	C7—C8	1.516 (3)
O4—C12	1.419 (3)	C7—H7A	0.9700
O4—C10	1.421 (3)	C7—H7B	0.9700
N1—C1	1.348 (2)	C8—C9	1.512 (3)
N1—C3	1.371 (2)	C8—H8A	0.9700
N1—C6	1.479 (2)	C8—H8B	0.9700
N2—C1	1.345 (2)	C9—C10	1.520 (3)
N2—C4	1.372 (2)	C9—H9A	0.9700
N2—C5	1.468 (2)	C9—H9B	0.9700
C1—C2	1.524 (3)	C10—H10	0.9800
C3—C4	1.349 (3)	C11—H11A	0.9600
C3—H3	0.9300	C11—H11B	0.9600
C4—H4	0.9300	C11—H11C	0.9600
C5—H5A	0.9600	C12—H12A	0.9600
C5—H5B	0.9600	C12—H12B	0.9600
C5—H5C	0.9600	C12—H12C	0.9600
			
C10—O3—C11	112.8 (2)	C6—C7—H7A	108.6
C12—O4—C10	114.0 (2)	C8—C7—H7B	108.6
C1—N1—C3	109.36 (15)	C6—C7—H7B	108.6
C1—N1—C6	127.21 (16)	H7A—C7—H7B	107.6
C3—N1—C6	123.40 (15)	C9—C8—C7	112.45 (19)
C1—N2—C4	109.59 (15)	C9—C8—H8A	109.1
C1—N2—C5	126.70 (15)	C7—C8—H8A	109.1
C4—N2—C5	123.63 (15)	C9—C8—H8B	109.1
N2—C1—N1	106.65 (15)	C7—C8—H8B	109.1
N2—C1—C2	126.40 (16)	H8A—C8—H8B	107.8
N1—C1—C2	126.86 (16)	C8—C9—C10	114.3 (2)
O1—C2—O2	128.94 (19)	C8—C9—H9A	108.7
O1—C2—C1	115.65 (17)	C10—C9—H9A	108.7
O2—C2—C1	115.39 (17)	C8—C9—H9B	108.7
C4—C3—N1	107.34 (16)	C10—C9—H9B	108.7
C4—C3—H3	126.3	H9A—C9—H9B	107.6
N1—C3—H3	126.3	O3—C10—O4	112.1 (2)
C3—C4—N2	107.05 (16)	O3—C10—C9	107.4 (2)
C3—C4—H4	126.5	O4—C10—C9	112.8 (2)
N2—C4—H4	126.5	O3—C10—H10	108.1
N2—C5—H5A	109.5	O4—C10—H10	108.1
N2—C5—H5B	109.5	C9—C10—H10	108.1
H5A—C5—H5B	109.5	O3—C11—H11A	109.5
N2—C5—H5C	109.5	O3—C11—H11B	109.5
H5A—C5—H5C	109.5	H11A—C11—H11B	109.5
H5B—C5—H5C	109.5	O3—C11—H11C	109.5
N1—C6—C7	111.91 (15)	H11A—C11—H11C	109.5
N1—C6—H6A	109.2	H11B—C11—H11C	109.5
C7—C6—H6A	109.2	O4—C12—H12A	109.5
N1—C6—H6B	109.2	O4—C12—H12B	109.5
C7—C6—H6B	109.2	H12A—C12—H12B	109.5
H6A—C6—H6B	107.9	O4—C12—H12C	109.5
C8—C7—C6	114.69 (18)	H12A—C12—H12C	109.5
C8—C7—H7A	108.6	H12B—C12—H12C	109.5

### Hydrogen-bond geometry (Å, º)

**Table:**

*D*—H···*A*	*D*—H	H···*A*	*D*···*A*	*D*—H···*A*
C3—H3···O2^i^	0.93	2.30	3.141 (2)	151
C4—H4···O1^ii^	0.93	2.30	3.127 (2)	148

Symmetry codes: (i) *x*, *y*+1, *z*; (ii) *x*+1, *y*, *z*.
